# Long-Term Cognitive and Language Outcomes at the Age of Seven Following Arterial Presumed Perinatal Ischemic Stroke: A Case Report

**DOI:** 10.3390/brainsci15121291

**Published:** 2025-11-29

**Authors:** Ivana Bogavac, Ljiljana Jeličić, Jelena Đorđević, Maša Marisavljević, Nenad Polomac, Ivana Pavković, Mile Vuković

**Affiliations:** 1 Cognitive Neuroscience Department, Research and Development Institute “Life Activities Advancement Institute”, 11000 Belgrade, Serbia; lj.jelicic@add-for-life.com (L.J.); m.marisavljevic@add-for-life.com (M.M.); i.pavkovic@add-for-life.com (I.P.); 2 Department of Speech, Language and Hearing Sciences, Institute for Experimental Phonetics and Speech Pathology, 11000 Belgrade, Serbia; 3 Clinic for Neurology and Psychiatry for Children and Adolescents, 11000 Belgrade, Serbia; jelena.djordjevic@medf.kg.ac.rs; 4 Department of Psychiatry, Faculty of Medical Sciences, University of Kragujevac, 34000 Kragujevac, Serbia; 5 Institute of Neuroradiology, University Hospital Frankfurt am Main, 60596 Frankfurt am Main, Germany; 6 Faculty of Special Education and Rehabilitation, University of Belgrade, 11000 Belgrade, Serbia; mvukovic.dr@gmail.com

**Keywords:** cognition, language, arterial perinatal stroke, MRI

## Abstract

The brain in healthy adults shows language localization in the left hemisphere, and the evidence from the literature supports neural plasticity after traumatic injuries. What happens if an injury occurs early in brain development? How does early unilateral brain damage affect a child’s ability to acquire language? Evidence regarding language development after early unilateral brain damage is mixed. Therefore, this case report aims to present the language and cognitive status at the age of seven in a child who suffered a left-sided arterial presumed perinatal ischemic stroke (APPIS), with reference to her MRI findings. As part of her ongoing rehabilitation, she has received continuous speech therapy since age four and physiotherapy since six months of age. The current evaluation provides insights into long-term neurodevelopmental outcomes following early brain injury, highlighting the variability in clinical outcomes and considering the potential for functional restitution.

## 1. Introduction

Arterial presumed perinatal ischemic stroke (APPIS), with a prevalence of approximately 1:7900 [1], is classified among prenatal strokes. In contrast to neonatal arterial ischemic stroke (NAIS), neonatal cerebral sinovenous thrombosis (NCSVT), and neonatal hemorrhagic stroke (NHS)—which typically present with acute clinical symptoms—APPIS is characterized by delayed clinical presentation. It is therefore grouped together with periventricular venous infarction (PVI) and presumed perinatal hemorrhagic stroke [2]. Despite the late onset of symptoms and delayed diagnosis, APPIS usually involves cortical structures and consequently leads to more severe clinical outcomes than other forms of prenatal stroke. The most commonly reported initial symptom is unilateral spastic cerebral palsy (USCP) [3,4,5,6,7]. Owing to cortical involvement, epilepsy is also frequent, often accompanied by language and cognitive impairments [4,5,8].

The underlying mechanism typically affects an arterial vascular territory, most commonly the middle cerebral artery (MCA) [5,9]. The MCA and its branches supply the lateral surfaces of the temporal, parietal, and frontal lobes, as well as subcortical structures including the caudate nucleus, basal ganglia, and internal capsule [10]. Early left-hemispheric injury can lead to atypical right-hemisphere representation of language [11,12,13]. However, some studies suggest that language may remain predominantly intra-hemispheric rather than shifting inter-hemispherically [14]. These variations are most likely related to the timing and extent of the stroke, as well as the presence or absence of epileptic seizures [15,16,17].

In addition to evident motor deficits, neurodevelopmental impairments are among the most frequently reported consequences of APPIS and commonly include difficulties in cognition, language, and behavior, although numerous inconsistencies have been noted in the literature [18]. Variability in developmental timing, assessment methods, participant age, follow-up duration, and small sample sizes all contribute to these inconsistent findings.

Existing studies on global intelligence quotient (IQ) generally indicate that most children attain scores within the normal range [19,20,21], with lower scores more often observed in those with epilepsy or in children who experience frequent and severe postnatal seizures. The presence of seizures may further disrupt learning and knowledge acquisition by causing additional functional brain injury, thereby contributing to reduced intellectual performance [22,23,24].

Although many studies [25,26,27,28,29,30] have investigated neural plasticity and language reorganization following early brain injury, detailed descriptions of language abilities specifically in children with APPIS remain limited. Reported rates of language impairment vary substantially across studies, ranging from 20 to 25% [2,4] to 49–50% [31,32].

## 2. Case Presentation

### 2.1. Case Report

This study presents a case report of a child with a history of APPIS. In our previous case report [33], we described her cognitive and speech-language status at the age of five. The present report represents a continuation of that case, providing a qualitative follow-up and longitudinal case description at the age of seven. The current evaluation employed a different set of tests compared to the previous assessment. A direct comparison with previous results was performed only for the cognitive scores, while other outcomes, including language measures, are reported separately. The report emphasizes developmental changes and qualitative patterns in cognitive, language, and psychological outcomes over time. Specifically, it provides an updated assessment at the age of seven, focusing on her cognitive and language outcomes, as this age represents an important stage in the consolidation of speech and language. At the time of the study, she was the third child of healthy, non-consanguineous parents, living with her older brother and sister in a small town in the Republic of Serbia. Her family is monolingual, and her mother tongue is Serbian. She has been in the kindergarten since she was three. At the age of three years and ten months, she was brought by her parents to the Institute for Experimental Phonetics and Speech Pathology (IEPSP) in Belgrade, Republic of Serbia, for assessment and subsequent therapy. The first examination included an interview with parents, a review of medical records, observation of the child during free play and structured task activities, and the administration of diagnostic instruments. The anamnestic data were obtained from her medical documentation (the Labour and Delivery note, the reports by the child’s neurologist, physiatrist, and radiologist). She was born at term, after a spontaneous delivery, with an Apgar score of 9/10 and a birth weight of 3350 g. No complications were noted at birth or during the perinatal period.

When she was five months old, she was taken for a paediatric consultation because her parents noticed that she did not use her right arm and did not open her right fist. She was further referred for a neurology consultation, after which an MRI scan was performed. Consequently, at the age of six months, she was diagnosed with a cerebral infarction-massive perinatal ischemic stroke, predominantly affecting the territory of the left MCA. Further assessment included a standard electroencephalogram (EEG), which showed spike waves in the left frontal-central-temporal (FCT) region during the awake state. During deep non-rapid eye movement (NREM) sleep, the EEG revealed unilateral left-sided paroxysms accompanied by transient post-paroxysmal attenuation of the basic activity. Across follow-up evaluations conducted at later ages, the EEG findings were inconsistent but consistently indicated left-hemispheric epileptiform activity. Specifically, frequent medium-amplitude spike–wave complexes were recorded over the left central-parietal-temporal (CPT) region, occasionally spreading to homologous right-hemispheric areas, suggesting a left CPT epileptiform focus. In subsequent assessments, medium-amplitude biphasic sharp waves and spike–wave discharges were observed over the left parietal-temporal-occipital (PTO) region, predominantly during sleep. Later recordings revealed biphasic sharp waves over the same CPT region, occurring at a frequency of 2–3 Hz and only rarely propagating to the contralateral side. Consequently, the child did not receive antiepileptic medication and was regularly monitored by a neurologist. Genetic analysis showed that she was heterozygous for MTHFR C677T. Her motor development was delayed, supported with physiotherapy. At 18 months, she was diagnosed with USCP, and she began walking at 22 months. By this time, the family was advised to start speech and language therapy, in addition to continuing physiotherapy.

At the time of admission, she was three years and ten months old. She was minimally verbal, using up to ten functional words, and uncooperative; she also exhibited shame and negativism. She had severe receptive and expressive language deficits. Her comprehension was limited to simple verbal instructions used in daily routines, and her speech production was limited to single words, which she used to express needs or to name objects or actions. Syntax development was at an early stage; she used one to two word utterances with early grammar such as (/daj vodu/ meaning “give me water”, /mama, ajde/ meaning “mommy let’s go”). After initial observation and analysis of medical documentation, speech and language therapy was initiated to maximise developmental progress, considering all aspects of the child’s overall development. Kostic’s selective auditory filter amplifier (KSAFA) approach was the therapy of choice because it can be adjusted according to the individual needs and specifics of each child [33,34,35]. From the age at which she was first enrolled in therapy, the child participated in treatment continuously, three times per week for 60 min per session. During this period, she demonstrated consistent progress and underwent evaluations every twelve months by child psychologists and speech and language therapists. Finally, at the age of seven, she underwent a follow-up MRI scan. Here, we present her cognitive, speech, and language development at this age, after three years of therapy, in comparison with the MRI findings.

In summary, following an APPIS, the child was diagnosed with USCP and delayed speech and language development. She has received speech therapy since the age of four and physiotherapy since six months of age. This case report provides an updated assessment of this child at the age of seven, illustrating the longitudinal trajectory of her cognitive, speech, and language development during a period of emerging neurocognitive and language consolidation. The report also aims to highlight the variability in clinical outcomes and to underscore the significance of continuous therapy, with reference to her individual MRI findings, while considering the potential for functional restitution and its implications for optimizing individualized rehabilitation strategies.

### 2.2. Data Collection

Considering that the child was included in speech and language therapy at the age of four, she underwent regular assessments throughout this period. The assessments were adjusted to the child’s chronological age and included different instruments over time. At seven years of age, she underwent a follow-up assessment and a control MRI scan. The cognitive, speech, and language assessment was conducted in the morning in a quiet room, with the child seated opposite the examiner. The MRI scan was performed under general anaesthesia and interpreted by an experienced neuroradiologist.

A cognitive assessment was performed by the child’s clinical psychologist. The instrument of choice was REVISK [36] which is a revised Wechsler Intelligence Scale for Children in Serbian. According to Wechsler’s principle, the test is standardized for measuring children’s overall intellectual functioning and cognitive abilities. The instrument provides insight into total, verbal, and performance scores, where higher scores reflect higher levels of intellectual functioning. The Verbal and Performance Scales consist of 5 subtests, where Information, Comprehension, Arithmetic, Similarities, and Digit Span belong to the Verbal Scale subtests, and Picture Completion, Picture Arrangement, Block Design, Object Assembly, and Coding belong to the Performance Scale subtests.

Speech and language assessment was performed by an experienced speech therapist with 20 years of clinical practice. The assessment was performed during three sessions in the morning, in a quiet room without interruptions. The instruments of choice included the New Reynell Developmental Language Scales—Serbian version (NRDLS-SR), a revised and standardised version for the Serbian language [37]; the Comic Strip Story [33,38,39]; the Peabody Picture Vocabulary Test (PPVT) [40,41]; the Global Articulation Test [41,42,43]; the Test for Analytical estimation of Serbian Sounds [33,42]; and the Oral Praxis Test [33,42].

The NRDLS-SR [37] is a standardized test instrument used for the assessment of language development in children, evaluating both receptive language (comprehension) and expressive language (production) abilities. The Comprehension scale includes a total of 72 tasks, divided into eight sections, while the Production scale consists of 64 tasks across seven sections. The Comprehension scale assesses a child’s understanding of individual words (nouns and verbs), two-word utterances, predicates and their complements in simple sentences, verb morphology, and pronouns (reflexive and non-reflexive clitics). It also evaluates comprehension of complex syntactic structures, such as relative clauses, sentences with noncanonical word order, and negative sentences. Finally, it evaluates inferential reasoning based on contextual information. The Production scale assesses a child’s ability to produce individual words, including nouns and verbs, two-word utterances, and predicates with their complements in simple sentences. It also evaluates verb morphology, production of complex syntactic structures, including questions, relative clauses, passive sentences, and sentences with noncanonical word order. Furthermore, it evaluates grammaticality judgment, assessing the child’s ability to determine the grammatical correctness of sentences. Together, these scales provide a comprehensive evaluation of a child’s vocabulary, grammatical knowledge, sentence construction, and higher-order language skills.

The Comic Strip Story [39] is used for the elicitation of narrative. The event sequence is represented with four pictures in the form of a comic strip. Based on the sequence of pictures examinee is supposed to form a short story without any guided questions or a model given. The examinee’s statement is recorded, later in the process of data processing, it is transcribed and analysed for its structure. The PPVT [40] is an instrument for the assessment of receptive vocabulary, specifically the comprehension of spoken words. The test consists of pictures that the examinee has to match with words that he heard. It includes names of objects, actions, or phenomena. The Global Articulation Test is a screening tool for the assessment of sound production and types of errors in the phonetic inventory of Serbian, for more details, see Bogavac et al. [42]. The Test for Analytical Estimation of Serbian Sounds assesses sound production and types of errors in more detail, taking into consideration specific sound characteristics, for more see Bogavac et al. [33]. The Oral Praxis Test [33,42] is a tool for assessment of motor imitation ability for the orofacial region. It includes simple and complex movements of the jaw, tongue, and lips. The examiner demonstrates the movement, and the child is supposed to do the same.

The complete study protocol followed Ethical Principles in Medical Research Involving Human Subjects, which is established by the Declaration of Helsinki. Furthermore, the study was approved by the Ethics Committee of the Institute for Experimental Phonetics and Speech Pathology (No C-22-01; Date: 10 February 2022) in Belgrade, Serbia. This approval has included the parents’ written consent for participation in the study as well as cognitive, speech and language assessments.

## 3. Results

### 3.1. MRI Scan Results 

**Figure 1:**
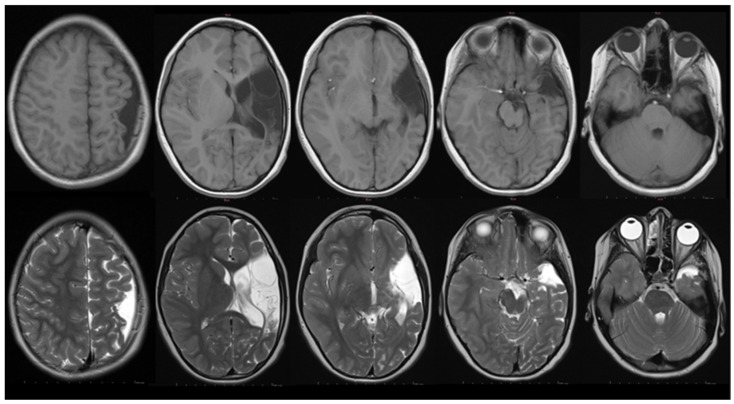
MRI scan: T1 (**upper row**) and T2 (**lower row**) weighted MRI images of the patient in the axial plane. The columns show the following axial levels: the central region (**first column**), basal ganglia (**second column**), thalami (**third column**), mesencephalon (**fourth column**), and pons (**fifth column**).

The child suffered an ischemic stroke affecting the entire left MCA territory. (Figure 1) There is extensive parenchymal damage involving pars opercularis and triangularis of the inferior frontal gyrus, known as Broca’s area. Additionally, the superior temporal gyrus and supramarginal gyrus (Wernicke’s region), along with the arcuate fasciculus, are also affected by the infarction. Caudally, there is clear evidence of Wallerian degeneration involving the thalamus, mesencephalon, and pons ipsilateral. Cranially, at the level of the central region, the precentral gyrus (M1 territory) and postcentral gyrus (S1 territory) were found to be rudimentary. One can observe prominent compensatory growth in the right hemisphere with normal white and gray matter MRI signal characteristics. Furthermore, asymmetrical skull growth is noted, corresponding to the increasing volume of the right hemisphere and lagging development of the left hemisphere.

### 3.2. Cognitive Assessment Results

Results of the cognitive assessment are presented in Table 1.

At 5 years of age, the child exhibited a marked discrepancy between her Verbal IQ and Performance IQ, resulting in a disharmonious cognitive profile characterized by weaker verbal abilities. Specifically, her performance on the verbal scale fell within the below-average range, whereas her performance on the performance scale corresponded to average intellectual functioning. Additionally, a pronounced inter-test scatter was observed within both scales.

On the other hand, at 7 years of age, the cognitive assessment shows a balanced cognitive status, with no discrepancy between verbal and performance IQ, and both fall within the average range. Furthermore, inter-subtest scatter has become less pronounced, suggesting an overall evening-out of global verbal and performance cognitive abilities.

Within the verbal scale, Similarities remains the weakest area, currently at −1 SD, while within the performance scale, Block Design remains her weakest subtest, currently at −1 SD.

### 3.3. Speech and Language Assessment Results

The child’s comprehension and production scores on NRDLS-SR, which illustrate her abilities across various language domains, are summarized in Table 2.

Analysis of the Comprehension scale revealed that the child demonstrated successful performance in understanding individual words, including nouns and verbs, and as two-word utterances in which attention is directed toward semantic relationships and the expression of spatial relations. She also achieved adequate results in comprehending predicates and their complements within simple sentences. However, deficits were observed in verb morphology, specifically in understanding the use of the present tense, with 4 out of 6 correct responses. Pronouns, including two referential forms (reflexive and non-reflexive clitic pronouns), were correctly identified in 5 out of 6 responses. Difficulties were also noted in complex syntactic structures, including comprehension of relative clauses, sentences with noncanonical word order, and negative clauses, with 8 out of 10 correct responses. Inferential reasoning was slightly below expectations, with 8 out of 10 correct responses.

On the Production scale, performance was adequate in tasks involving the production of individual words, including nouns and verbs, and two-word utterances, in which attention is also directed toward semantic relationships and the expression of spatial relations, similar to the Comprehension scale. Nevertheless, deficits were evident in the production of predicates and their complements in simple sentences (8 out of 10 correct), verb morphology, specifically in the use of tense (present tense) (3 out of 6 correct), and complex syntactic structures, including questions, relative clauses, passive constructions, and sentences with noncanonical word order, where only 3 out of 12 responses were correct. Additionally, performance on grammaticality judgment, which evaluates the ability to assess the grammatical correctness of a sentence, was below average, with 10 out of 14 correct responses.

Overall, her receptive language abilities corresponded approximately to a developmental age of 5 years and 6 months, while her expressive language abilities corresponded to a developmental age of 5 years and 2 months, approximately two years below her chronological age.

The Comic Strip Story—she successfully specified characters, the place of action, and marked the outcome. She correctly followed the chronological sequence of events; however, she was unable to express the causality of events or to draw a conclusion. She used short, informative sentences with correct grammar. She produced two sentences containing four words, one sentence with three words, and two sentences consisting of only two words. The sentences included nouns, pronouns, verbs, and prepositions. Nouns and pronouns were used in grammatically appropriate forms with respect to case, gender, and number. Verbs were correctly inflected for both the present and past tense. However, she demonstrated difficulties in organising her thoughts and formulating them into coherent verbal statements.

On the PPVT, she achieved a standardized score of 91, which placed her in a low category. It corresponds to an age equivalent of 6 years and 4 months, indicating that her receptive vocabulary skills are slightly below her chronological age of seven years.

The Global Articulation Test and the Test of Analytic Estimation of Serbian Sounds indicated that she had not yet acquired all 30 speech sounds in her phonetic inventory. She used a substitute sound /r/ without adequate vibration at the language tip for the trill /r/; the lateral /l/ she pronounced interdentally (it refers to inadequate place of pronunciation—it is an alveolar sound); and she pronounced three sounds /ʥ͡, z, ʐ/ slightly devoiced.

The Oral Praxis Test revealed six movements that she was unable to imitate and three movements that she could perform partially. All of these involved distinct patterns of tongue movement.

## 4. Discussion

This case report presents the neurocognitive and language status at age seven in a child with a history of APPIS, who sustained a massive, unilateral cortical-subcortical insult primarily in the territory of the left middle cerebral artery. She has received speech therapy since age four and physiotherapy since six months of age. Her cognitive and speech-language profile at age five was previously described [33], providing context for her developmental trajectory. The current findings offer an updated assessment during a key period of speech and language consolidation. These results are discussed in relation to her MRI findings and the existing literature on neurodevelopmental outcomes following early unilateral brain injury, providing a comprehensive context for interpretation. To address the inconsistent evidence on neurocognitive and language development in children with perinatal stroke, we aimed to highlight the variability in clinical outcomes and underscore the importance of continuous therapy in supporting long-term neurodevelopment.

### 4.1. MRI Scan Findings

In this case, the ischemic injury encompassed the entire left MCA territory, including key language-related regions such as Broca’s area (pars opercularis and triangularis of the inferior frontal gyrus), Wernicke’s region (superior temporal and supramarginal gyri), and the arcuate fasciculus. Such extensive lesions are associated with variable cognitive and language outcomes, consistent with previous reports of perinatal arterial ischemic stroke [44,45,46,47,48]. The presence of Wallerian degeneration extending caudally to the thalamus, mesencephalon, and pons ipsilateral aligns with a prior study linking these changes with long-term neuromotor outcome and may be a predictor of motor impairment [49]. Notably, the prominent compensatory growth observed in the contralesional right hemisphere, with normal white and gray matter MRI signal characteristics, underscores the brain’s capacity for neuroplastic reorganization following unilateral perinatal lesions. The asymmetrical skull growth, corresponding to increased right hemisphere volume and delayed development of the left hemisphere, further highlights how structural remodelling may be reflected not only in cortical architecture but also in cranial morphology. This phenomenon has been well-documented in the literature, highlighting the brain’s adaptability in response to early injury [47,50,51,52]. In addition, recent findings indicate widespread differences in cortical structure and volume within the contralesional hemisphere of children with perinatal arterial ischemic stroke, with more pronounced alterations compared to other lesion types. Such changes in cortical morphometry are thought to mediate residual functional capacity and may represent potential targets for neuroplastic interventions aimed at enhancing recovery and adaptive reorganization [53]. Furthermore, recent research indicates that the structural connectome of the contralesional hemisphere is altered after perinatal stroke and correlates with clinical function, emphasizing the role of the intact hemisphere in functional recovery [54]. These findings align with the concept that neuroplasticity, particularly in the contralesional hemisphere, plays a crucial role in supporting long-term neurodevelopmental outcomes in children with perinatal stroke. The observed structural alternations and their association with functional outcomes underscore the relevance of neuroplastic mechanisms in the evaluation and treatment of children with perinatal stroke. In addition, although prominent compensatory growth is observed in the right hemisphere, such structural reorganisation does not necessarily ensure full functional recovery. Specifically, the adaptive restitution of the right hemisphere may be incomplete or suboptimal, and even with evident morphological changes it may not fully support the integration required for typical language processing [55,56,57]. In our case, the MRI findings obtained at the age of seven were interpreted in the context of the observed long-term cognitive and language outcomes, suggesting a link between structural alterations and functional outcomes. The neuroplasticity and compensatory role of the right hemisphere in supporting cognitive and language functions are clearly evident, yet residual language deficits remain. These findings underscore the complex relationship between structural neuroplasticity and functional outcomes following early brain injury, reflecting significant progress alongside remaining deficits, all observed within the context of intensive therapeutic intervention and stimulation. Finally, they highlight the importance of considering both structural and functional measures when evaluating recovery and planning interventions in children with early brain injury.

Moreover, when interpreting the child’s developmental trajectory, electrophysiological findings offer an additional layer of context. In the present case, the child exhibited subclinical left-hemispheric epileptiform discharges, recorded without overt clinical manifestations. No clinical seizures were observed during follow-up, and consequently, the child did not receive antiepileptic medication. However, despite the absence of clinical seizures, these subclinical discharges may have contributed to residual language deficits [58,59,60], although their precise impact remains uncertain within the broader context of compensatory right-hemispheric neuroplasticity and intensive therapy.

### 4.2. Cognitive Status

Previous research indicates that perinatal arterial ischemic stroke can lead to long-term cognitive and behavioral difficulties that may persist throughout life [61]. More specifically, studies have shown that a greater extent of the stroke is associated with poorer neurodevelopmental outcomes and lower cognitive performance in children compared to normative samples, often resulting in low-average or borderline intelligence levels [45,62]. Additionally, bilateral infarcts have been linked to a 58% risk of cognitive impairment [50].

However, some studies suggest that although estimated IQ performance at the individual level may fall below the normal range, the mean IQ score for the overall sample does not significantly differ from the theoretical average of the normative population [63]. In our study, the findings indicate that at the age of seven, the child demonstrated cognitive functioning within the average range; her performance did not differ from the normative sample in Full Scale IQ, Verbal IQ, or Performance IQ. This outcome aligns with previous research showing that children with perinatal arterial ischemic stroke may exhibit normal intellectual functioning [63]. Considering the findings from our previous study conducted when the participant was five years old [33], and her current performance, these results further support the positive role of neuroplasticity and positive effects of the treatment.

Nevertheless, performance one standard deviation below the mean was observed on the Similarities subtest, suggesting persistent difficulties in higher-level language functions such as abstraction and verbal reasoning. Similarly, lower performance on the Block Design subtest indicates ongoing visuomotor difficulties, likely related to the primary neurological condition. These results are consistent with findings showing that children with perinatal arterial ischemic stroke may experience subtle, enduring cognitive challenges that persist through later developmental stages [61].

Moreover, longitudinal studies have reported that while preschool-aged children with perinatal arterial ischemic stroke may perform comparably to their peers in Full Scale, Verbal, and Performance IQ, school-aged children often demonstrate significantly lower scores in Full Scale IQ, Working Memory, and Processing Speed, though not in Verbal or Performance IQ [64]. These findings reveal a notable decline over time, reflecting emerging deficits in higher-order cognitive abilities during the school years. Therefore, continued monitoring is essential, as achieving average IQ levels at present does not necessarily guarantee the maintenance of typical cognitive performance in later stages of development.

### 4.3. Speech and Language Status

Children with perinatal arterial ischemic stroke frequently demonstrate heterogeneous language outcomes, ranging from near-typical development to persistent difficulties in both expressive and receptive language [18,27,46,65,66]. Moreover, such deficits may only become evident when higher-level linguistic or cognitive functions are assessed [22,65,67]. The NRDLS-SR results in this case report revealed below-age performance in both comprehension and expressive language abilities, and a clear discrepancy between the two domains. This pattern aligns with previous research, which demonstrates that language outcomes following perinatal stroke are often uneven, with comprehension more likely to be preserved than expressive abilities [22,66]. More specifically, in this case study, the delay in comprehension was milder compared to the more pronounced deficit in expressive language. The child’s receptive language abilities corresponded approximately to a developmental age of 5 years and 6 months. In contrast, expressive language abilities corresponded to a developmental age of 5 years and 2 months, reflecting a delay of approximately two years relative to chronological age. This profile is consistent with previous findings in children with perinatal left-hemispheric lesions, where receptive language tends to recover earlier and to a greater extent due to interhemispheric reorganization [26,68]. Furthermore, authors Lidzba et al. [68] suggest that, relative to chronological age, children with perinatal stroke may exhibit a gap of several years between expected and actual expressive language performance, with partial recovery often occurring by school age.

A detailed analysis of comprehension deficits revealed that, although the child retained the ability to understand individual words, two-word utterances, and simple sentences, notable difficulties were observed in processing verb morphology, particularly the use of the present tense, and in complex syntactic structures, including relative clauses, sentences with noncanonical word order, and negative clauses. Inferential reasoning was also slightly below expectations. This profile aligns with previous findings in children with perinatal left-hemispheric lesions, where receptive language is often relatively preserved while morphosyntactic processing and higher-order inferential skills present greater challenges [22,66,68].

On the other side, a detailed analysis of production deficits revealed that, although the child was able to produce individual words, including nouns and verbs, and two-word utterances reflecting basic semantic and spatial relationships, notable difficulties were observed in producing predicates and their complements in simple sentences, verb morphology, particularly tense (present tense), and complex syntactic structures, including questions, relative clauses, passive constructions, and sentences with noncanonical word order. Difficulties in expressive syntax and morphology observed in the current case suggest that long-term language outcomes depend not only on lesion location but also on the efficiency of compensatory mechanisms and continuous language stimulation. These findings align with studies reporting that even children who achieve age-appropriate comprehension scores may still exhibit deficits in higher-order linguistic processing and narrative organization [69,70]. Additionally, performance on grammaticality judgment, which assesses the ability to evaluate the correctness of sentences, was also below average, further highlighting the uneven trajectory of language development in this child.

In general, the observed profile reflects the uneven pattern observed across both comprehension and production, highlighting the greater vulnerability of morphosyntactic and complex expressive abilities compared with simpler lexical and semantic skills. Furthermore, this trajectory is consistent with literature indicating that, although basic language comprehension is often preserved, many children exhibit specific deficits in complex syntactic structures, morphology, and verbal fluency [69,71].

A detailed analysis of the Comic Strip Story task revealed that the child produced narratives with a limited number of words and word types. Syntactic complexity was very low, with few multiword sentences or varied sentence structures. Additionally, the narrative structure was poorly organized, with minimal sequencing of events and no causal connections between actions. Overall, these findings indicate significant difficulties in organizing and expressing ideas coherently during narrative production which is consistent with findings reported in the literature [69,72].

Based on the PPVT results, it is evident that although the child demonstrated understanding of individual words, her overall receptive vocabulary performance fell below the age-appropriate level. The recent study [55] reported that this discrepancy persisted in individuals assessed after 12 years of age following perinatal stroke, whereas it was not observed in younger children. Similar findings have been reported [72], showing that receptive vocabulary skills do not differ significantly between typically developing children and those who suffered prenatal stroke when assessed at younger ages.

Given the limited literature on oromotor performance in children with APPIS, this case report provides additional insight into specific oromotor and articulatory deficits associated with early left-hemispheric lesions. The child’s articulatory difficulties likely reflect impaired tongue and articulator control due to USCP following perinatal stroke. Her incomplete phonetic inventory and inability to perform specific oral praxis movements indicate disrupted coordination of precise tongue gestures required for accurate phoneme production. In this case, errors such as the absence of trill /r/, interdental realization of /l/, and partial devoicing of fricatives suggest reduced precision in tongue placement and movement timing. These observations may reflect subtle subclinical challenges in voluntary tongue and articulator control, consistent with her oromotor and phonetic profile.

The results of the language assessments indicate that, despite persistent deficits, early and targeted stimulation of language functions remains crucial for maximizing developmental potential in children with neurological impairments. Our findings support the importance of early, systematic intervention, particularly within frameworks that integrate individualized and multidisciplinary approaches. In this context, the KSAFA system provides a comprehensive therapeutic model grounded in the principles of individualization, coordinated multidisciplinary engagement, and dynamic reprogramming of treatment strategies. By synchronizing and optimizing multiple systems and functions, KSAFA system aims to facilitate the child’s progression toward their highest attainable level of functioning.

## 5. Conclusions

This case highlights the considerable variability in clinical, cognitive, and language outcomes following APPIS and the persistence of receptive and expressive language deficits, underscoring the limitations of compensatory neural mechanisms in achieving complete functional restitution. Behavioral observations, combined with structural MRI findings at age seven, suggest a clear link between lesion characteristics, including alterations in the contralesional hemisphere, and functional outcomes. Together, these findings emphasize the critical role of neuroplasticity in supporting uneven language recovery, illustrating how individual differences in lesion location, severity, and neural reorganization can shape developmental trajectories. Importantly, this case underscores the value of continuous, individualized therapeutic interventions, which may contribute to enhanced functional restitution and support long-term neurodevelopmental trajectories in the context of perinatal stroke.

## Figures and Tables

**Table 1:** Results of cognitive assessments.

	Subscales	5 Years		7 Years		
		Score (SD)	IQ	Score (SD)	IQ	Full Scale IQ
Verbal scale	Information	5 (/)	66	9 (/)	90	91
	Comprehension	3 (−1)		10 (/)		
	Arithmetic	5 (/)		8 (/)		
	Similarities	4 (/)		7 (−1)		
	Digit Span	6 (+1)		8 (/)		
Performance scale	Picture Completion	10 (/)	93	9 (/)	92	
	Picture Arrangement	11 (+1)		9 (/)		
	Block Design	5 (−2)		7 (−1)		
	Object Assembly	7 (−1)		10 (/)		
	Coding	12 (+2)		9 (/)		

Note. The Full-Scale IQ could not be calculated at 5 years of age due to the marked discrepancy between the child’s Verbal IQ and Performance IQ.

**Table 2:** Child’s Comprehension and Production Scores on the NRDLS-SR.

Section	Comprehension Scale	Section	Production Scale
Selecting Objects	10/10	Naming Objects	10/10
Relating Two Objects	10/10	Relating two objects	10/10
Verbs	10/10	Verbs	10/10
Sentence Building	10/10	Sentence building	8/10
Verb morphology	4/6	Verb morphology	3/6
Pronouns	5/6		
Complex sentences	8/10	Complex sentences	3/12
Inferencing	8/10	Grammar judgment	10/14

## Data Availability

The original contributions presented in this study are included in the article. Further inquiries can be directed to the corresponding author.

## Abbreviations

The following abbreviations are used in this manuscript:

APPIS	Arterial presumed perinatal ischemic stroke
NAIS	Neonatal arterial ischemic stroke
NCSVT	Neonatal cerebral sinovenous thrombosis
NHS	Neonatal hemorrhagic stroke
PVI	Periventricular venous infraction
USCP	Unilateral spastic cerebral palsy
MRI	Magnetic resonance imaging
MCA	Middle cerebral artery
IQ	Intelligence quotient
IEPSP	Institute for Experimental Phonetics and Speech Pathology
EEG	Electroencephalogram
FCT	Frontal-central-temporal
NREM	Non-rapid-eye movement
CPT	Central-parietal-temporal
KSAFA	Kostic’s selective auditory filter amplifier
REVISK	Serbian adaptation of the Wechsler Intelligence Scale for Children-Revised
NRDSL-SR	New Reynell Developmental Scale—Serbian version
PPVT	Peabody Picture Vocabulary Test
